# A *Tricholoma matsutake* Peptide with Angiotensin Converting Enzyme Inhibitory and Antioxidative Activities and Antihypertensive Effects in Spontaneously Hypertensive Rats

**DOI:** 10.1038/srep24130

**Published:** 2016-04-07

**Authors:** Xueran Geng, Guoting Tian, Weiwei Zhang, Yongchang Zhao, Liyan Zhao, Hexiang Wang, Tzi Bun Ng

**Affiliations:** 1 State Key Laboratory for Agrobiotechnology and Department of Microbiology, China Agricultural University, Beijing 100193, China; 2 Institute of Biotechnology and Germplasmic Resource, Yunnan Academy of Agricultural Science, Kunming 650223, China; 3 College of Food Science and Technology, Nanjing Agricultural University, Weigang, Nanjing 210095, China; 4 School of Biomedical Sciences, Faculty of Medicine, The Chinese University of Hong Kong, Shatin, New Territories, Hong Kong, China

## Abstract

Hypertension is a major risk factor for cardiovascular disease. A crude water extract of the fruiting bodies of a highly prized mushroom *Tricholoma matsutakei* exerted an antihypertensive action on spontaneously hypertensive rats (SHRs) at a dosage of 400 mg/kg. An angiotensin converting enzyme (ACE) inhibitory peptide with an IC_50_ of 0.40 μM was purified from the extract and designated as TMP. Its amino acid sequence was elucidated to be WALKGYK through LC-MS/MS analysis. The Lineweaver-Burk plot suggested that TMP was a non-competitive inhibitor of ACE. A short-term assay of antihypertensive activity demonstrated that TMP at the dosage of 25 mg/kg could significantly lower the systolic blood pressure (SBP) of SHRs. TMP exhibited remarkable stability over a wide range of temperatures and pH values. It also demonstrated 2,2-diphenyl-1-picrylhydrazyl (DPPH) radical scavenging activity. The aforementioned activities of TMP were corroborated by utilizing the synthetic peptide. Hence *T. matsutake* can be used as a functional food to help prevent hypertension- associated diseases.

Cardiovascular disease is one of the main causes of death worldwide, and hypertension (high blood pressure) is a leading risk factor for cardiovascular disease. According to a survey conducted by the World Health Organization, hypertension currently kills nine million people every year^1^. Good control of the blood pressure will have a great impact on the health status of human populations and will prevent cardiovascular disease^2^.

Angiotensin converting enzyme (ACE, EC.3.4.15.1), a Zn-metallopeptidase, plays a key role in the regulation of peripheral blood pressure mainly through the renin-angiotensin (RAS) and kallikrain-kinin systems (KKS)^3^. ACE catalyzes the conversion of inactive Angiotensin-I (Ang-I) to Angiotensin-II (Ang-II). Ang-II, a potent vasoconstrictor, stimulates the secretion of aldosterone, which enhances sodium and water re-absorption in the nephron, and therefore increases the arterial pressure by bringing about a rise in the intravascular fluid volume^4^. Thus, ACE has been considered as a target in the prevention and treatment of hypertensive diseases. A series of ACE inhibitors including captopril, enalapril, lisinopril and so on has been synthesized and currently used clinically as antihypertensive drugs^5^. These synthetic ACE inhibitors are believed to have various side effects such as cough, taste disturbances and skin rashes, which motivated researchers to develop novel, safe and natural ACE inhibitors from food-derived antihypertensive peptides as alternatives to synthetic drugs^6^.

Recently, many reports on bioactive natural ACE inhibitors from food sources, e.g., milk, turtle egg white, soybean, sweet potato etc have appeared^7^,^8^. ACE inhibitory peptides and proteins have also been successfully purified from edible mushrooms, such as *Grifola frondosa*^9^, *Pholiota adiposa*^10^, *Pleurotus cornucopiae*^11^, *Pleurotus cystidiosus*^12^, *Tricholoma giganteum*^13^, *Hypsizygus marmoreus*^14^, *Agaricus bisporus*^15^, *Leucopaxillus tricolor* and *Ganoderma lucidum*^16^.

*Tricholoma matsutake*, a well-known wild edible mushroom, is an ectomycorrhizal basidiomycete which grows in a symbiotic relationship with Pinaceae in the Northern hemisphere and produces prized “matsutake” mushroom^17^. The fruiting body of the pine mushroom is commercially important in the Far East as a valuable food, not only because of its attractive flavor but also because of the medicinal effects^18^. Many bioactive molecules have been isolated from *T. matsutake* such as nuclease^19^, polysaccharide^20^, laccase^21^ and α-galactosidase^22^. These substances with diverse biological activities are beneficial to human health and are useful in environmental protection.

The purpose of this study was to isolate and identify the ACE inhibitory peptide from *T. matsutake*, and to investigate the inhibitory effect of the purified ACE inhibitory peptide on the spontaneously hypertensive rats. This work would provide new evidence for further advocating *T. matsutake* as a functional food to prevent chronic diseases.

## Results

### Comparison of ACE inhibitory activities in water extracts from fruit bodies of mushrooms of the *Tricholoma* genus

Extracts from the fruit bodies of eight mushrooms that all belong to the genus *Tricholoma* were prepared for examination of their respective inhibitory activity on ACE. As shown in Table 1, the percentages of inhibition of ACE activity of the eight *Tricholoma* mushrooms were in the range of 2.4–95.0%, with the water extract of *T. matsutake* displaying the most potent inhibitory activity. This was followed by *T. mongolicum* and *T. saponaceum* extracts, which brought about 63.9% and 38.2% inhibition of ACE activity, respectively. The lowest ACE inhibitory activity (10.3% and 2.4% inhibition, respectively) was shown by extracts of *T. pessundatum* and *T. bakamatsutake Hongo*.

### Purification and identification of ACE inhibitory peptide from *T. matsutake*

**Figure 1:**
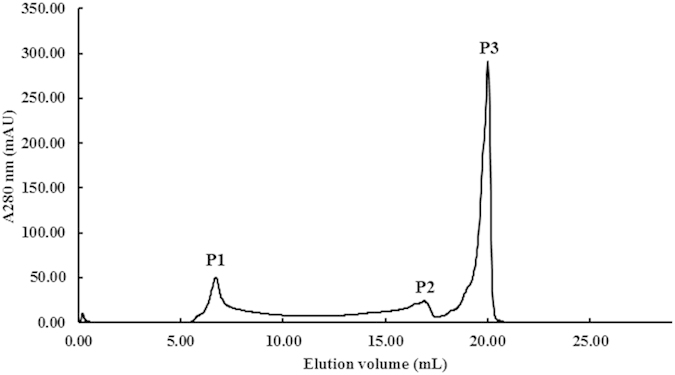
FPLC-gel filtration on Superdex Peptide 10/300 GL column. Eluent: distilled water; Fraction size: 0.8 mL; Flow rate: 0.5 ml/min. Fraction P3 represents purified *Tricholoma matsutake* ACE inhibitory peptide, designated as TMP.

The purification protocol entailed extraction with distilled water, ultrafiltration through a membrane, followed by two ion-exchange chromatography steps on Q-Sepharose and Mono-Q, respectively, and finally fast protein liquid chromatography (FPLC) on a Superdex Peptide column. The procedure was efficacious for purifying *T. matsutake* ACE inhibitory peptide. The water extract was subjected to ultrafiltration through a 5-kDa molecular weight cut-off membrane. Filtrate with a molecular weight (M.W.) over 5 kDa elicited 27% inhibition of ACE activity while filtrate with a M.W. below 5 kDa showed 63% inhibition of ACE activity. Thus the active filtrate with a M.W. below 5 kDa was employed for isolation of ACE inhibitory peptide. After a series of purification procedures, three peaks were observed upon FPLC-gel filtration on a Superdex Peptide 10/300 GL column. Among these peaks, fraction P3 expressed the strongest ACE inhibitory activity (Fig. 1).

Five peptides were obtained in P3 by linear trap quadrupole (LTQ) LC-MS/MS analysis (Table 2). To confirm the ACE inhibitory activity of these peptides, they were chemically synthesized. The chemically synthesized peptide WALKGYK designated as TMP exhibited the highest ACE inhibitory activity (IC_50_ = 0.40 μM) and was further studied.

### Antioxidant property (free radical scavenging activity) of ACE inhibitory peptide from *T. matsutake*

**Figure 2:**
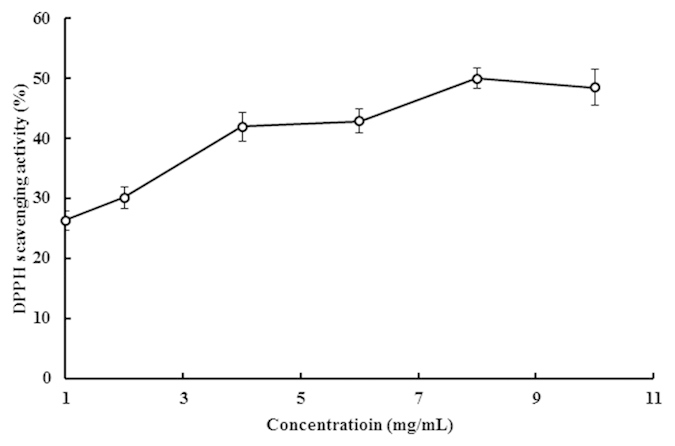
Scavenging capacity of *Tricholoma matsutake* ACE inhibitory peptide on DPPH radicals. Results represent mean ± SD (n = 3).

Interestingly, it was found that the isolated ACE inhibitory peptide TMP demonstrated DPPH radical scavenging activity (Fig. 2). In this study, the DPPH radical scavenging activity of TMP increased in a concentration-dependent manner. At a higher concentration (10 mg/mL), 50% of the DPPH radicals were scavenged. TMP exhibited a dose-dependent scavenging action on DPPH radicals in the range of concentrations studied^23^.

### Effects of pH and temperature on ACE inhibitory activity of peptide from *T. matsutake*

**Figure 3:**
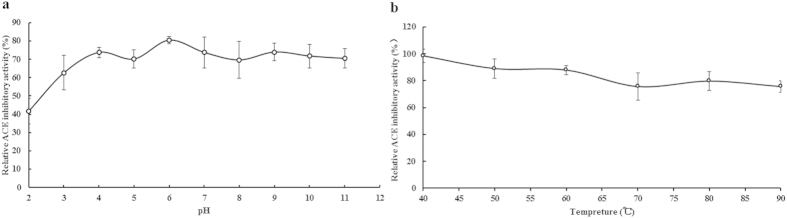
Effects of pH and temperature on the ACE inhibitory activity of *Tricholoma matsutake* ACE inhibitory peptide. (**a**) Effect of pH on TMP. (**b**) Effect of temperature on TMP. Results represent mean ± SD (n = 3).

As shown in Fig. 3, the isolated inhibitory peptide TMP retained ACE inhibitory activity after exposure to different pH values and different temperatures for 2 hours. TMP displayed good thermostability and pH stability.

### Mode of inhibition of ACE inhibitory peptide from *T. matsutake*

**Figure 4:**
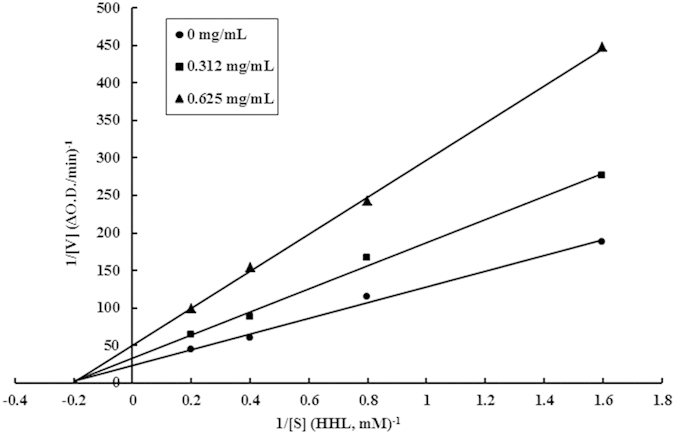
Lineweaver-Burk plot. Effect of *Tricholoma matsutake* ACE inhibitory peptide (TMP) on ACE: (⚫) control, 0 mg TMP/mL, (◼) 0.312 mg TMP/mL, (▲) 0.625 mg TMP/mL.

The mode of inhibition of the isolated inhibitory peptide WALKGYK on ACE was ascertained from the Lineweaver-Burk plot. According to the plot presented in Fig. 4, which showed ACE activity in either the presence or absence of 0.325 mg/mL or 0.675 mg/mL TMP, the three straight lines intersected at the same point on the horizontal axis of the graph plotting 1/S, indicating that the same Km was attained regardless of inhibitor concentration, whereas the maximum velocity differed. The findings indicate that TMP was a non-competitive inhibitor of ACE^24^.

### Antihypertensive action of the purified ACE inhibitory peptide from *T. matsutake*

**Figure 5:**
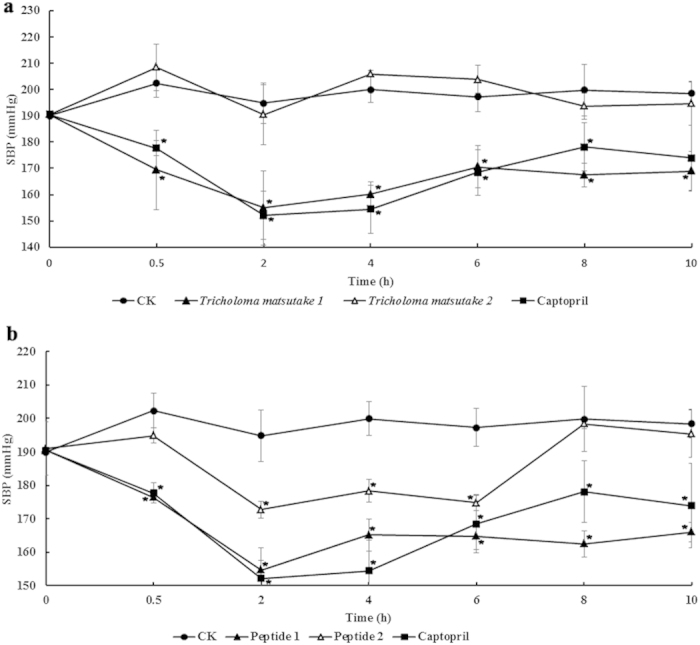
Changes in systolic blood pressure (SBP) in spontaneously hypertensive rats after oral administration of a water extract of *Tricholoma matsutake* and purified ACE inhibitor TMP. (**a**) Single oral administration was performed with a dosage of 200 mg water extract/kg body weight and 400 mg water extract/kg body weight, respectively. SBP was measured 0, 0.5, 2, 4, 6 and 8 h after administration. (*)Significantly different from control at *p* < 0.05 by student’s *t*-test. (●) control, 0.9% saline solution; (◼) captopril; (Δ) 200 mg water extract/kg body weight; (▴) 400 mg water extract/kg body weight. (**b**) Single oral administration was performed with a dosage of 25 mg TMP/kg body weight and 50 mg TMP/kg body weight, respectively. SBP was measured 0, 0.5, 2, 4, 6 and 8 h after administration. (*)Significantly different from control at *p* < 0.05 by student’s *t*-test. (●) control, 0.9% saline solution; (▪) captopril; (▵) 25 mg TMP/kg body weight; (▴) 50 mg TMP/kg body weight.

The antihypertensive action of the isolated inhibitory peptide TMP was investigated by measuring the change of SBP in SHRs at 0.5, 2, 4, 6 and 8 h after oral administration of TMP as shown in Fig. 5a,b. The average blood pressure of the SHRs in the test groups was 190 mmHg just before the oral administration. Half an hour after the oral administration of captopril (100 mg/kg body weight [BW]), in the inhibitory peptide-treated groups (25 and 50 mg/kg BW) as well as the crude extract-treated group (400 mg/kg BW), the blood pressure had undergone a slight decrease. There were significant differences in SBP after treatment with the inhibitory peptide, the high dose of crude extract and the control 2 h after oral administration. The maximum decrease in SBP value caused by 100 mg/kg BW of captopril, 25 and 50 mg/kg BW of the inhibitory peptide and 400 mg/kg BW of the crude extract was observed at 2 h, and the decrements in SBP produced were 38 mm Hg, 18 mm Hg, 36 mm Hg and 36 mm Hg, respectively. The blood pressures increased gradually 4 h after oral administration in all groups. The peptide group of 50 mg/kg BW exerted an antihypertensive effect equipotent to the positive group until 8 h following administration. There was no significant difference in SBP between rats receiving 200 mg/kg BW of the crude extract and the control rats. Furthermore, no allergic reactions or coughing was noted on the day of the experiment and on the following day.

## Discussion

The present account represents the first report on the isolation of the peptide WALKGYK from the renowned edible mushroom *T. matsutake* which inhibited ACE with an IC_50_ value of 0.4 μM and exhibited a clear antihypertensive effect on spontaneously hypertensive rats. A tripeptide GQP from *T. giganteum* belonging to the same genus manifested ACE inhibitory activity^13^.

There is a paucity of information about the constituents of mushrooms belonging to the genus *Tricholoma*. Hence the ACE inhibitory activities of eight mushrooms belonging to the genus of *Tricholoma* were examined in the present study (Table 1). There was a large disparity among the different *Tricholoma* species regarding ACE inhibitory activity, signifying that ACE inhibitory activity was not correlated with the genus. It was noted that the water extract of *T. matsutake* had potent ACE inhibitory activity.

At present, more and more studies are focused on bioactive peptides like ACE inhibitory peptides. The bioactive peptides from mushrooms documented for reducing blood pressure are listed in Table 3. The highest ACE inhibitory activity was shown by peptides from *Pholiota adiposa*^10^, *Grifola frondosa*^9^ and *T. giganteum*^13^ with the lowest IC_50_ values which were 0.1, 0.12 and 0.13 μM, respectively. The peptide isolated from *T. matsutake* in the current study and peptide from *Hypsizygus marmoreus*^14^ exhibited a lower IC_50_ value, which were a little higher than the peptide from *T. giganteum* but substantially lower than other mushroom peptides, including peptides from *Pleurotus cystidiosus*^12^ and *Agaricus bisporus*^15^.

Food-derived peptides with antihypertensive activity have been identified mostly based on their ability to inhibit ACE *in vitro*^2^. Potent ACE inhibitory peptides are usually composed of 2–12 amino acid residues^25^, which contain aromatic or hydrophobic residues at the C-terminus, positively charged amino acids in the intermediate domain, and hydrophobic amino acids residues such as alanine, proline, tyrosine, and valine residues at the N-terminus^26^. This is in accordance with the findings of Rohrbach *et al*. that, the most potent ACE inhibitors always contain hydrophobic amino acid residues at the three C-terminal positions^27^. Peptide TMP contained four hydrophobic amino acid residues (representing an abundance of 57%), and one of the tyrosines was present in the C-terminal tripeptide sequence. Moreover, in a previous study, the amino acids proline and lysine or aromatic amino acid residues appeared in most naturally occurring ACE inhibitory peptides^28^. Peptide TMP contained two aromatic amino acid residues (tryptophan and tyrosine) and the amino acid residue lysine, in keeping with the characteristic feature of naturally occurring ACE inhibitory peptides.

It was disclosed by many researchers that peptides with ACE inhibitory activity usually possessed some antioxidant activity, such as DPPH radical scavenging activity at the same time. A native peptide RPSYT in commercial soybean infant formulas has both antioxidant activity and antihypertensive properties^29^. Antioxidant and ACE inhibitory peptides have been extracted and isolated from two different types of Thai traditional fermented shrimp pastes^30^. The peptide TMP isolated in the present study exhibited high ACE inhibitory activity and concurrently DPPH radical scavenging effect. On account of the relationship between hypertension and oxidant stress, these active peptides expressing both antihypertensive and antioxidant activities merit further investigations. They may have considerable potential as multifunctional ingredients in functional foods for controlling chronic diseases.

The isolated peptide TMP was a non-competitive ACE inhibitor. Non-competitive inhibitors have been located in *Agaricus bisporus*^15^, *Hypsizygus marmoreus*^14^ and *P. cornucopiae*^11^ as shown in Table 3. In recent years, some other food sources containing peptidic non-competitive ACE inhibitors have been isolated, for example, peptides from cuttlefish^31^ and peptide from fertilized eggs^32^. Unfortunately, the relationship between the mode of inhibition and structure of these peptides has not been fully unravelled^24^.

ACE inhibitory activity *in vitro* is not always directly related to an antihypertensive effect *in vivo*^33^. Thus, demonstration of an *in vivo* antihypertensive effect is of paramount importance, and there have been many investigations on the antihypertensive effect of edible mushrooms and other foodstuff on SHRs. The peptide TMP identified from *T. matsutake* manifested a significant antihypertensive action on SHRs, which may be beneficial to hypertensive patients and advocates the peptide TMP as a candidate for development into an antihypertensive drug. The water extract of *T. matsutake* (400 mg/kg) engendered a 19% decline in blood pressure in SHRs from 190 mm Hg to 154 mm Hg while the water extract of *H. marmoreus* at the dosage of 800 mg/kg lowered the blood pressure by 14.4%, less potently than *T. matsutake*. The water extract of *P. coruncopiae* displayed a more pronounced effect on suppressing the blood pressure than *T. matsutake* and *H. marmoreus*. There are many bioactive substances for lowering the blood pressure encompassing various peptides, phenolic compounds, proteins, polysaccharides, saponins, sterols, pigments, fiber as well as minerals^34^, which may explain why the IC_50_ values of ACE inhibitory activity of peptides from *H. marmoreus* and *T. matsutake* were almost identical while the crude water extract of *T. matsutake* was superior to *H. marmoreus* in reducing blood pressure^14^. The dosage of the crude extract of *T. matsutake* was 400 mg/kg, which corresponded to 14.5 g of fruiting bodies of *T. matsutake*. The peptide TMP from *T. matsutake* evinced good thermostability, which ensures preservation of the ACE inhibitory activity when *T. matsutake* is employed in tea or other food items. All this may furnish evidence for *T. matsutake* being a functional food to help prevent hypertension- associated diseases.

## Materials and Methods

Fresh fruiting bodies of the mushroom *Tricholoma matsutake* were collected from Yunnan Province in China. Q-Sepharose, Mono-Q, Superdex peptide 10/300 GL and AKTA Purifier were purchased from GE Healthcare. Hippuryl-L-histidyl-L-leucine (HHL), 2,2-diphenyl-1-picrylhydrazyl(DPPH), and phenyl-methylsulfonyl fluoride (PMSF) were purchased from Sigma-Aldrich (St.Louis, MO, USA). All solvents and chemicals used in this study were of analytical and HPLC grade.

Twelve-week-old male spontaneously hypertensive rats (SHRs) were purchased from Beijing Vital River Experimental Animal Technical co., Ltd.

### Assay of ACE inhibitory activity

ACE was prepared using fresh rabbit lungs according to the protocol reported by Hayakari *et al*.^35^ with some modifications. After washing with ice-cold 0.15 M NaCl, the rabbit lungs without connective tissues were cut up to small pieces and homogenized in 10 volumes of ice-old 0.1 M sodium borate buffer (pH 8.3) containing 0.25 M sucrose and 0.1 mM PMSF. The homogenate was centrifuged at 9,000 rpm for 40 min at 4 °C after dialysis at 4 °C overnight in 0.1 M sodium borate buffer (pH 8.3). The resulting supernatant was centrifuged at 9,000 rpm for 40 min at 4 °C again. The ACE activity in the supernatant was determined and the supernatant was aliquoted and then stored at −20 °C for further experiments.

The ACE inhibitory activity was assayed by using a spectrophotometric method following the protocol described by Cushman^36^ with modifications. Briefly, an aliquot (20 μL) of the sample was mixed with 20 μL ACE and 150 μL 5 mM HHL in 0.1 M borate buffer (pH 8.3) containing 0.3 M NaCl. The reaction mixture was incubated at 37 °C for 30 min, and then the reaction was terminated by adding 500 μL 1 M HCl. The hippuric acid formed was extracted with 1.2 mL ethyl acetate and centrifuged at 4,000 rpm for 10 min. Then 800 μL of the upper organic phase was heat-evaporated at 95 °C for 35 min to remove ethyl acetate. The dried residue was dissolved in 800 μL distilled water and vortex mixed, and the absorbance was measured spectrophotometrically at 228 nm to estimate ACE inhibitory activity. ACE-inhibitory activity was calculated by using the equation 1:


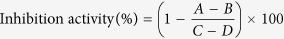


where A was the absorbance of the solution containing ACE and inhibitor, B was the absorbance of solution containing ACE and inhibitor but terminated in advance, C was the absorbance of the solution containing ACE without inhibitor, and D was the absorbance of the solution containing ACE without inhibitor but terminated in advance. The concentration of the inhibitor required to inhibit 50% of the ACE activity under the above assay conditions was defined as IC_50_.

### Purification of ACE inhibitor

Fresh fruiting bodies of the mushroom *T. matsutake* (stored at −20 °C) were soaked in distilled water (weight: volume = 1:2) at 4 °C for 1 hour, and then homogenized in a Waring blender. The homogenate was extracted overnight at 4 °C before centrifugation at 9,000 rpm for 20 min at 4 °C. The supernatant was centrifuged again at 9,000 rpm for 20 min after boiling for 10 min. Then the supernatant was subjected to ultrafiltration with a 5 kDa M.W. cut-off membrane. The filtrate and solution of the filter-cake were assayed for ACE inhibitory activity as described above. The active fraction was applied on an anion exchange Q-Sepharose column (2.5 cm × 20 cm) in Tris-HCl buffer (10 mM, pH 8.8) with a flow rate of 2 mL/min. After removal of the unadsorbed fraction Q1, adsorbed material was fractionated using a linear concentration gradient of 0–1 M NaCl in the same buffer. The fraction with ACE inhibitory activity was loaded on another strong anion exchange Mono-Q column (4.6 cm × 10 cm) in Tris-HCl buffer (10 mM, pH 8.0) with a flow rate of 1 mL/min. Following removal of the unadsorbed fraction, the column was further eluted with a linear gradient of 0–1 M NaCl in the same buffer. Fractions with ACE inhibitory activity were pooled, and final purification by fast protein liquid chromatography (FPLC) on a Superdex peptide gel filtration column at a flow rate of 0.8 mL/min using an AKTA Purifier was carried out. The most active fraction was lyophilized for further research.

### Identification and synthesis of the purified peptide

After lyophilization, the most active fraction was dissolved in 0.1% formic acid and 2% acetonitrile for liquid chromatography-tandem mass spectrometry (LC-MS/MS) analysis using a LTQ-Orbitrap mass spectrometer (Thermo Electron, Bremen, Germany). Samples were loaded onto a reversed-phase C18 column with a diameter of 0.1 mm × 10 cm and a particle size of 3 μm. Separation of the peptides was achieved by using a gradient of 5 to 35% acetonitrile in 0.1% formic acid over 120 min. The acquired MS/MS data were transformed to MGF by BIWORKS, and the data were searched against the NCBI database using MASCOAT software.

To investigate antihypertensive activity on SHRs, antioxidant capacity and mode of inhibition of ACE, the purified peptide was synthesized by Shanghai Bootech BioScience & Technology Co., Ltd. The purity of the synthetic peptide was 99% as evidenced by HPLC analysis. The IC_50_ value of the peptide was investigated according to the method described above.

### Antioxidant property (free radical scavenging activity)

The DPPH radical scavenging activity of the inhibitor was determined according to the method described by Cheng *et al*.^23^ with slight modifications. An aliquot of 200 μL of the inhibitor at different concentrations (1–10 mg/mL) was mixed with 600 μL 0.004% DPPH dissolved in methanol. The mixture was left at room temperature in the dark for 30 min. Scavenging capacity was measured spectrophotometrically by monitoring the decrease in absorbance at 517 nm. The DPPH radical scavenging activity was calculated by using the equation 2:


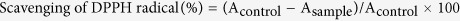


where A_control_ was the absorbance in the absence of sample, A_sample_ was the absorbance in the presence of the sample.

### Effects of pH and temperature on ACE inhibitory activity

The inhibitory peptide was dissolved in distilled water and subsequently incubated for 2 h at different pH values ranging from 2 to 11. The pH value was then adjusted to 7.0. ACE inhibitory activity was determined with the abovementioned method.

The inhibitory peptide was incubated for 2 h at different temperatures covering a range from 40 °C to 90 °C. After acclimation to room temperature, the ACE inhibitory activity was assayed at 37 °C as mentioned above.

### Determination of mode of inhibition of ACE

The mode of inhibition of ACE by the peptide was determined spectrophotometrically using HHL as the substrate to distinguish between competitive, non-competitive and uncompetitive inhibition. Basically, a series of concentrations (0.63, 1.25, 2.5 and 5 mM) of HHL were incubated with ACE in the presence or absence of the peptide (0, 0.312 and 0.625 mg/mL). The ACE inhibitory activity was determined according to the aforementioned method. The kinetic parameters Vmax (maximum velocity) and Km (Michaelis constant) were investigated by constructing Lineweaver-Burk plots.

### Antihypertensive action in spontaneously hypertensive rats

All experiments were performed in accordance with the Regulations of Experimental Animal Administration issued by the State Committee of Science and Technology of the People’s Republic of China and also with the approval of the Animal Experimentation Ethics Committee of The Chinese University of Hong Kong which had been procured prior to the animal experiments. SHRs with systolic blood pressure (SBP) higher than 180 mmHg were randomly divided into six groups after acclimation for 1 week. During the acclimatization period prior to the test, the blood pressures of the rats were measured four times weekly. Each group containing four rats. A blank control group receiving oral administration of 0.9% saline solution and a positive control group receiving oral administration of captopril (100 mg/kg body weight) were included. The ACE inhibitory peptide-treated group received the peptide at two different concentrations: 25 mg/kg body weight and 50 mg/kg body weight. The group treated with crude water extract of *T. matsutake* received the extract at two different concentrations: 200 mg/kg body weight and 400 mg/kg body weight. SBP was measured thrice at each of the following time points: 0, 0.5, 2, 4, 6 and 8 h after drug/sample administration, using a tail-cuff method with a programmable BP-100A electro-sphygmomanometer (Chengdu Taimeng Software Co., Ltd., Chengdu, China).

## Additional Information

**How to cite this article**: Geng, X. *et al*. A *Tricholoma matsutake* Peptide with Angiotensin Converting Enzyme Inhibitory and Antioxidative Activities and Antihypertensive Effects in Spontaneously Hypertensive Rats. *Sci. Rep.* **6**, 24130; doi: 10.1038/srep24130 (2016).

## Figures and Tables

**Table 1:** ACE inhibiting activities of water extracts of *Tricholoma* mushrooms^a^.

Mushroom Species	ACE Inhibitory Activity (%) of Water Extract
*Tricholoma matsutake*	95.0 ± 0.2
*Tricholoma terreum*	35.3 ± 0.4
*Tricholoma pessundatum*	10.3 ± 0.1
*Tricholoma mongolicum*	63.9 ± 0.5
*Tricholoma saponaceum*	38.2 ± 0.7
*Tricholoma bakamatsutake Hongo*	2.4 ± 0.4
*Tricholoma giganteum*	29.7 ± 0.2
*Tricholoma myomyces*	19.6 ± 0.0

^a^The ratio of mushroom to distilled water (weight to volume) used for preparing the extracts was 1:2. Values were means ± S.D. of three determinations.

**Table 2:** ACE inhibitory activity of peptides from *Tricholoma matsutake* a.

Peptide	IC_50_(μM)
LLVTLKK	0.95 ± 0.12
IISKIK	1.19 ± 0.26
ILSKLK	4.02 ± 0.31
LIDKVVK	0.62 ± 0.42
WALKGYK	0.40 ± 0.29

^a^Values were means ± S.D. of three determinations.

**Table 3:** ACE inhibitory peptides from mushrooms of different genera.

Mushroom species	Peptide	Mode of inhibitory of ACE	IC_50_ (μM)
*Tricholoma matsutake*	WALKGYK	Non-competitive	0.40
	AHEPVK	Competitive	63
*Agaricus bisporus*^15^	RIGLF	Competitive	116
	PSSNK	Non- competitive	129
*Tricholoma giganteum*^13^	GQP	Competitive	0.13
*Pleurotus cystidiosus*^12^	AHEPVK	Competitive	62.8
	GPSMR	Competitive	277.5
*Hypsizygus marmoreus*^14^	LSMGSASLSP	Non-competitive	0.33
*P. cornucopiae*^11^	RLPSEFDLSAFLRA	Competitive	0.28
	RLSGQTIEVTSEYLFRH	Non-competitive	0.56
*Pholiota adiposa*^10^	GQGGP	ND	0.1
*Grifola frondosa*^9^	VIQKYP	Competitive	0.12

“ND” means “Not Detected”.
